# Molecular Simulation Study on the Interaction between Porcine CR1-like and C3b

**DOI:** 10.3390/molecules28052183

**Published:** 2023-02-26

**Authors:** Zhen Hou, Wei Yin, Zhili Hao, Kuohai Fan, Na Sun, Panpan Sun, Hongquan Li

**Affiliations:** 1 Shanxi Key Lab for Modernization of TCVM, College of Veterinary Medicine, Shanxi Agricultural University, Jinzhong 030801, China; 2 College of Veterinary Medicine, Jilin University, Changchun 130015, China

**Keywords:** CR1-like, C3b, immune adhesion, molecular dynamics, molecular docking

## Abstract

The molecular basis of porcine red blood cell immune adhesion function stems from the complement receptor type 1-like (CR1-like) on its cell membrane. The ligand for CR1-like is C3b, which is produced by the cleavage of complement C3; however, the molecular mechanism of the immune adhesion of porcine erythrocytes is still unclear. Here, homology modeling was used to construct three-dimensional models of C3b and two fragments of CR1-like. An interaction model of C3b–CR1-like was constructed by molecular docking, and molecular structure optimization was achieved using molecular dynamics simulation. A simulated alanine mutation scan revealed that the amino acids Tyr761, Arg763, Phe765, Thr789, and Val873 of CR1-like SCR 12–14 and the amino acid residues Tyr1210, Asn1244, Val1249, Thr1253, Tyr1267, Val1322, and Val1339 of CR1-like SCR 19–21 are key residues involved in the interaction of porcine C3b with CR1-like. This study investigated the interaction between porcine CR1-like and C3b using molecular simulation to clarify the molecular mechanism of the immune adhesion of porcine erythrocytes.

## 1. Introduction

The immune function of erythrocytes has been extensively studied in biology and medicine. Studies have shown that erythrocytes have an immune adhesion function, the molecular basis of which is complement receptor type 1 (CR1), which is present on the surface of erythrocyte membranes. One ligand of CR1 is C3b, which is formed by the cleavage of serum complement C3. Serum complement C3 is activated by antigens or immune complexes (IC) and is cleaved into C3a and C3b. C3b can then bind with antigens or IC to form C3b–antigen or C3b–IC. Erythrocytes specifically bind C3b via CR1 to transport the antigen or IC to the reticuloendothelial system for clearance and to inhibit complement overactivation [1]. When the amount or viability of erythrocyte CR1 decreases, IC is excessively deposited in tissues, contributing to severe tissue damage and a variety of immune diseases, such as rheumatoid arthritis [2], lupus erythematosus [3], and Alzheimer’s disease [4]. Therefore, the alteration of erythrocyte immune adhesion function is highly correlated with disease onset, progression, regression, and immune status [5]. Veterinary studies have shown that erythrocytes of non-primate animals, such as pigs and chickens, have immune adhesion functions [6]. A variety of diseases are accompanied by changes in erythrocyte immune adhesion function, such as avian influenza [7], infectious bursal disease [8], and Marek’s disease [9]. We found that the molecular basis of the immune adhesion function of porcine erythrocytes is complement receptor type 1-like (CR1-like) on the surface of porcine erythrocyte membranes [10], which binds to C3b to exert its immune adhesion function [11]. Porcine erythrocyte CR1-like promotes the capture of sensitized GFP-Escherichia coli by porcine alveolar macrophages through its immune adhesion function under in vitro coincubation conditions [12]. These results suggest that porcine erythrocyte CR1-like may have an immune-presenting function. After, antigens or IC activate the porcine complement system and bind to C3b, C3b–antigen or C3b–IC binds porcine erythrocyte CR1-like and is transferred to macrophages for clearance [13,14]. However, the molecular mechanism by which CR1-like mediates the immune adhesion function of porcine erythrocytes is still unclear and needs further investigation.

CR1 belongs to the regulators of complement activation (RCA) family and is a type I transmembrane glycoprotein. In humans, CR1 is thought to have four major allelic variants, and the most common and well-studied type of CR1 has an extracellular region composed of 30 short consensus repeat (SCR) sequences [15]. The 30 SCR domains are arranged into four long homologous repeat (LHR) regions, designated LHR-A to LHR-D, each consisting of seven SCR domains, followed by two C-terminal SCR domains. The SCR 8–10 and SCR 15–17 domains of human CR1 are binding sites for C3b, and the sequences of SCR 8–10 and SCR 15–17 are almost identical. The SCR domains that make up CR1 are also known as complement control proteins (CCP) or sushi sequences [16]. CR1 and other members of the RCA family are almost entirely composed of 4–30 SCR domains. These proteins include membrane cofactor protein (MCP, CD46), factor H (fH), C4b-binding protein (C4BP), decay accelerating factor (DAF, CD55), and others. In addition to being present in the RCA family, SCR modules are also found in several other proteins in the complement system that interact with C3b/C4b. These include C1r, C1s, C2, factor B, mannose-binding lectin-associated serine proteases (MASP) 1, MASP 2, C6, and C7. All of these contain two or three SCR modules. Each SCR module consists of approximately 60 residues, each with a compact hydrophobic core that is wrapped in a β-sheet framework that is connected by two strictly conserved disulfide bonds. Although SCR modules have a conserved backbone, residue insertions and substitutions are common, and conservation between different SCR domains ranges from 20% to 100%. At least 25 SCR modules have been resolved, of which 14 SCR modules are from human RCA family proteins. When all resolved SCR modules are superimposed onto the SCR 16 domain of the factor H, the equivalent Cα RMSD values range from 1.9 Å to 3.1 Å [17].

As a core component of the complement system, human C3 is cleaved into β and α chains before being released from cells, which are covalently linked by disulfide bonds. The native molecular weight of C3 is 185 kDa, with the β chain being approximately 75 kDa and the α chain being approximately 110 kDa. The native C3 protein is composed of 13 distinct structural domains, and a series of proteolytic reactions lead to the removal of small peptide segments or entire structural domains, giving rise to various C3 fragments. C3 is sequentially cleaved into C3a (9 kDa), C3b (176 kDa), C3c (139 kDa), and C3d (34 kDa) through a series of proteolytic events, all of which occur within the α chain while the β chain remains unchanged. C3b is able to bind with various RCA proteins to exert regulatory effects, and the co-crystal structures of C3b–MCP SCR 1–4, C3b–CR1 SCR 15–17, C3b–DAF SCR 2–4, and C3b–FH SCR 1–4 have been resolved. These co-crystal structures reveal that RCA proteins and C3b adopt a common binding mode [18].

Research on the binding mode between CR1 and C3b has mainly focused on humans, and although it has been verified through yeast two-hybrid experiments that porcine C3b can bind to CR1-like SCR 19–21 [19], the lack of corresponding crystal structures has hindered further research. Recent advances in the structure of human CR1 and C3b, particularly the resolution of the human C3b–CR1 co-crystal structure (PDB ID:5FO9) [20], provide a reference for simulating the binding of porcine CR1-like to C3b, and this study will provide a new approach to elucidate the molecular mechanism of porcine red blood cell immune adhesion function.

## 2. Results

### 2.1. Sequence and Binding Interface Analyses

The protein sequences of human CR1 and porcine CR1-like were separately imported into SMART, and the normal mode was selected to determine the amino acid numbers and domain distributions of the two sequences. SMART analysis showed that the porcine CR1-like CDS region contains 2079 amino acids, which are composed of 31 short consensus repeat sequences (SCRs). Porcine CR1-like SCR 19–21 was shown to interact with C3b through yeast two-hybrid experiments. Studies on human CR1 have shown that SCR 8–10 and SCR 15–17 participate in the interaction with human C3b, and the sequences of SCR 8–10 and SCR 15–17 are identical. The alignment of human CR1 SCR 15–17 with porcine CR1-like sequences showed that porcine CR1-like SCR 12–14 (60% similarity) and SCR 19–21 (75% similarity) are the two most similar segments to human CR1 SCR 15–17 (Figure S1A,B). Among the 31 SCR domains in porcine CR1-like, SCR 19–21 has the highest similarity with, and an identical function to, human CR1 SCR 15–17 and may therefore be the equivalent segment of human CR1 SCR 15–17. The second most similar segment to human CR1 SCR 15–17 is porcine CR1-like SCR 12–14, which is separated from porcine CR1-like SCR 19–21 by 4 SCR domains, consistent with the sequence of human CR1. Therefore, porcine CR1-like SCR 12–14 may be the equivalent segment of human CR1 SCR 8–10. An analysis of the signal peptides and transmembrane helices of porcine C3b, porcine CR1-like SCR 12–14, and SCR 19–21 protein sequences showed that these sequences do not contain signal peptides or transmembrane helices (Figure S2). 

To further investigate whether the binding mode of human CR1-C3b and porcine CR1-like–C3b is similar, it is necessary to confirm the conservation of the binding interface, which is a prerequisite for subsequent work. Therefore, we first analyzed the binding interface of the human C3b–CR1 SCR 15–17 complex crystal structure (PDB ID: 5FO9). The amino acids of the human C3b–CR1 SCR 15–17 complex binding interface are shown in Table S1. By comparing the residues of the human C3b–CR1 SCR 15–17 binding interface with the equivalent residues of porcine C3b, CR1-like SCR 12–14, and SCR19–21, the results showed that over 70% of the human C3b residues on the binding interface were conserved in porcine C3b. For human CR1 residues on the binding interface, over 50% of the residues were conserved in porcine CR1-like SCR 12–14 and over 70% of the residues were conserved in porcine CR1-like SCR 19–21 (Figure S3). The hotspots of the binding interface were predicted using the PredHS, KFC, and DrugScore^PPI^ servers (Table S2). For human C3b hotspot residues, all other hotspot residues except Val762 were conserved in porcine C3b. Among the hotspot residues of human CR1 SCR 15–17, Tyr978, Arg980, Thr1006, Asn1012, Arg1082, Val1090, and Tyr1114 were conserved in porcine CR1-like SCR 12–14, and Tyr978, Thr1006, Asp1009, Asn1012, Met1014, Val1017, Arg1082, Val1090, and Tyr1114 were conserved in porcine CR1-like SCR 19–21 (Figure S3). This indicates that the binding interface of human CR1–C3b is conserved in the equivalent segments of porcine, and it is feasible to construct a model of the interaction between porcine CR1-like and porcine C3b based on the crystal structure of the CR1–C3b complex. 

### 2.2. Homology Modeling and Evaluation

**Figure 1:**
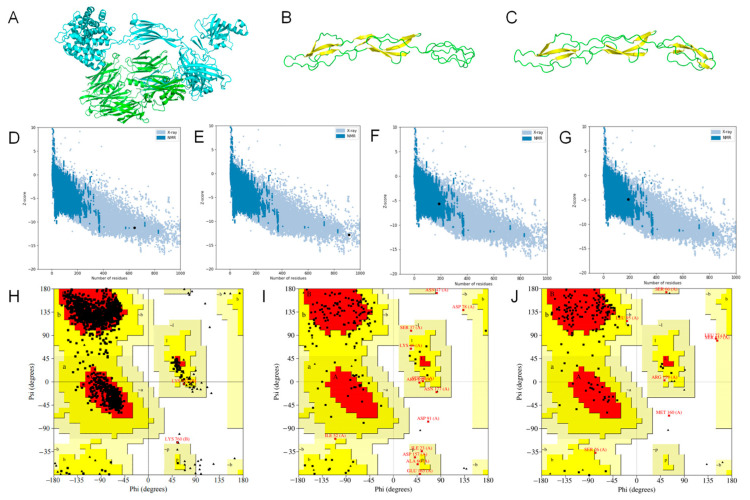
Homology modeling and assessment of C3b and CR1-like fragments. (**A**) C3b model constructed by AlphaFold-Multimer. The green chain is the C3b β chain, and the cyan chain is the C3b α chain. (**B**) CR1-like SCR 12–14 model constructed by I-TASSER. The yellow region is the β-fold, and the green region is the loop region. (**C**) CR1-like SCR 19–21 model constructed by I-TASSER. The yellow region is the β-fold, and the green region is the loop region. (**D**–**G**) Z scores of C3b β chain, C3b α chain, CR1-like SCR 12–14, and CR1-like SCR 19–21. (**H**–**J**) Ramachandran plots of C3b, CR1-like SCR 12–14 and, CR1-like SCR 19–21. Glycine residues are displayed as triangles while other residues are displayed as squares. Residues in the most favored regions (A, B, L) are those with phi and psi angles that are most energetically favorable. Residues in additional allowed regions (a, b, l, p) are less energetically favorable, but still allowed. Residues in generously allowed regions (~a, ~b, ~l, ~p) are the least energetically favorable but still possible.

The 3D structure model of porcine C3b was constructed using AlphaFold-Multimer. The five best models generated were evaluated according to the local distance difference test and predicted alignment error scores. The model with the highest confidence level after relaxation was used as the final conformation of porcine C3b (Figure 1A). The alignment of the porcine C3b model with the human C3b crystal structure resulted in an RMSD value of 1.53 Å, indicating that the model structure is very similar to the human C3b crystal structure. Three-dimensional structural models of porcine SCR 12–14 (Figure 1B) and SCR 19–21 (Figure 1C) were constructed using I-TASSER. To assess the model quality, each of the generated models was evaluated by I-TASSER and given a confidence score (C-score), typically from −5 to 2, based on the importance of the thread template arrangement and convergence parameters for the structural assembly simulations. Higher scores indicate higher confidence in the model. For the model with the highest confidence, its expected template modeling score (TM-score) and root mean square deviation (RMSD) values of the atomic positions are also given. Models with a TM-score greater than 0.5 were considered to represent the true topology, while models with a TM-score of less than 0.17 were considered to have random similarity. The C-score, TM-score, and estimated RMSD values for the SCR 12–14, and SCR 19–21 models are shown in Table S3.

The three final models were validated using ProSA, PROCHECK, and Verify3D. ProSA detection evaluates a model from an energy perspective. The results of the ProSA test showed that the Z score values of C3b beta chain, C3b alpha chain, CR1-like SCR 12–14 and SCR 19–21 were−11.22, −11.95, −5.61, and −4.68, respectively, which were in the normal range (Figure 1D–G). Verify3D mainly examines whether the template sequence and the structure of the model match, and the test requires that at least 80% of amino acid residues have a compatibility score value greater than 0.2. The Verify3D results for the three structures showed that all three models had more than 80% of residues with scores greater than 0.2 (Table 1), and all passed the test. PROCHECK mainly evaluates the plausibility of the amino acid conformation; if the ratio of the favored area’s residues exceeds 90%, the structure is considered to have a good stereochemical structure. The PROCHECK results showed that the homology model structure of C3b had 92.2% and 7.7% of residues located in the favored and allowed regions, respectively, indicating good model quality (Figure 1H). The percentages of residues located in the favored and allowed regions were 50.6% and 40.9% for SCR 12–14 (Figure 1I) and 72.2% and 23.4% for SCR 19–21 (Figure 1J), respectively, indicating that the modeling quality of the porcine CR1-like-related fragments was poor and that further optimization of the structure was needed.

### 2.3. Model Optimization and Evaluation

**Figure 2:**
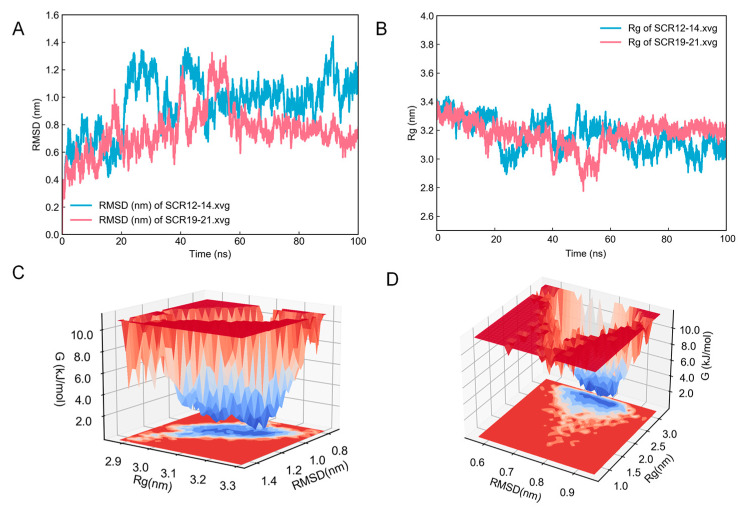
Molecular dynamics simulation and principal component analysis of CR1-like. (**A**) Backbone RMSD of CR1-like SCR 12–14 and CR1-like SCR 19–21. (**B**) Rg of CR1-like SCR 12–14 and CR1-like SCR 19–21. (**C**,**D**) PCA-based free energy landscape of CR1-like SCR 12–14 and CR1-like SCR 19–21.

**Figure 3:**
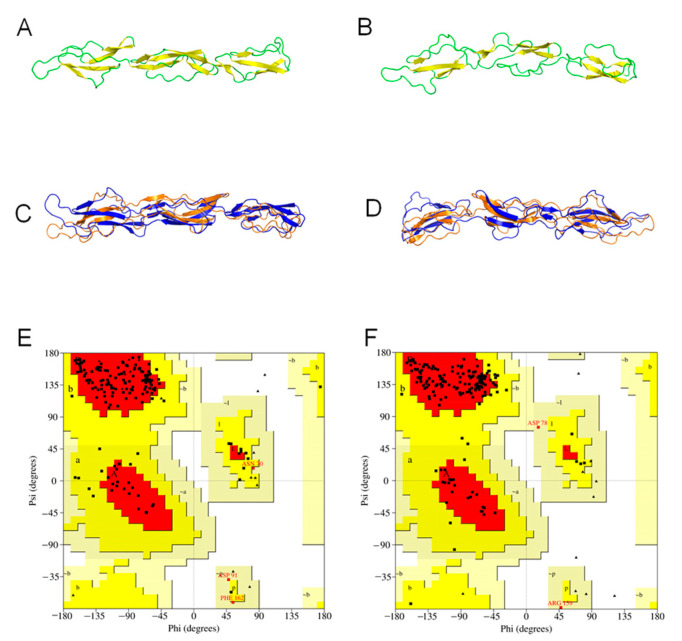
Protein structure optimization of CR1-like fragments. (**A**,**B**) CR1-like SCR 12–14 and CR1-like SCR 19–21 models after molecular dynamics simulation. The yellow region is the β-fold, and the green region is the loop region. (**C**,**D**) Protein conformational changes of CR1-like SCR 12–14 and CR1-like SCR 19–21 before and after molecular dynamics simulation. Orange models are the conformations before optimization, and blue models are the optimized conformations. (**E**,**F**) Ramachandran plots of optimized CR1-like SCR 12–14 and CR1-like SCR 19–21. Glycine residues are displayed as triangles while other residues are displayed as squares. Residues in the most favored regions (A, B, L) are those with phi and psi angles that are most energetically favorable. Residues in additional allowed regions (a, b, l, p) are less energetically favorable, but still allowed. Residues in generously allowed regions (~a, ~b, ~l, ~p) are the least energetically favorable but still possible.

A 100 ns molecular dynamics simulation was performed to refine the CR1-like structures generated by homology modeling. We analyzed the obtained MD trajectories to study the parameters RMSD and radius of gyration (Rg). As is shown in Figure 2A, during the simulation, the RMSD value of the SCR 12–14 configuration stabilized at approximately 1 nm after 50 ns. The RMSD value of the SCR 19–21 configuration stabilized at approximately 0.75 nm after 60 ns. The Rg of the target proteins reached a constant value after approximately 60 ns and showed a decreasing trend in the simulation, indicating that the proteins became more compact after the MD simulation (Figure 2B). Figure 2C,D shows the free energy landscapes (FELs) constructed via the RMSD and Rg values of the MD trajectories. The SCR 12–14 and SCR 19–21 models (Figure 3A,B) were the most stable protein structures extracted from the lowest energy wells of the free energy landscape map. 

Compared with the initial structures, the RMSD values of CR1-like SCR 12–14 and CR1-like SCR 19–21 after molecular dynamics simulation were 4.11 Å and 6.80 Å, respectively. The changes in the protein structures are shown in Figure 3C,D. The PROCHECK test showed that the residues located in the favored and allowed regions of the CR1-like SCR 12–14 model changed from 50.6% and 40.9% to 85.7% and 12.3% (Figure 3E), and the residues located in the favored and allowed regions of the CR1-like SCR 19–21 model changed from 72.2% and 23.4% to 88.0% and 10.8% (Figure 3F), respectively, indicating that the dynamic simulation significantly improved the 3D structures of both CR1 fragments (Table 1). The Verify3D results for the two structures showed that all models had more than 80% of residues with scores greater than 0.2, and all passed the test. The refined structures were found to be fit for further docking studies.

The CR1-like SCR 12–14 models generated by I-TASSER were structurally optimized and compared to models generated by trRosetta, RoseTTAFold, and AlphaFold, with resulting RMSD values of 2.11 Å, 1.67 Å, and 1.79 Å, respectively. For CR1-like SCR 19–21, the RMSD values were 2.53 Å, 3.18 Å, and 2.59 Å. Upon superimposing the structures, it was found that the extension direction of the backbone was essentially the same, the folding patterns in most regions were similar, and only some loop regions differed (Figure S4A,C). This indicates that there is not a significant difference in the structures of the CR1-like models generated by different tools.

The above structures were also compared to the crystal structure of human CR1 SCR 15–17. The modeling results from I-TASSER, trRosetta, RoseTTAFold, and AlphaFold were superimposed on the crystal structure of human CR1-like SCR15–17 (Figure S4B,D), and their RMSD values were calculated. For CR1-like SCR 12–14, the RMSD values were 2.49 Å, 2.62 Å, 2.06 Å, and 2.19 Å, respectively. For CR1-like SCR 19–21, the RMSD values were 1.85 Å, 2.16 Å, 2.49 Å, and 1.95 Å, respectively. SCR19–21 had a smaller RMSD than SCR12–14, which was expected because SCR19–21 is more similar to human CR1 in terms of its sequence. Overall, the models generated by different tools had similar RMSD values, indicating that the above models were similar to the crystal structure of human CR1.

The structural evaluation of the above models is shown in Table S4. The PROCHECK results for each model were similar, but the Verify 3D results for the model generated by I-TASSER after optimization were significantly better than those for the other models. Therefore, the model generated by I-TASSER was selected for subsequent molecular docking.

### 2.4. Molecular Docking and Dynamics Simulation

The optimized porcine C3b model was molecularly docked to the optimized porcine CR1-like SCR 12–14 and CR1-like SCR 19–21 using the ClusPro, ZDOCK, FRODOCK, HADDOCK, and GRAMM-X online servers. Free docking and residue-restrained docking were used to obtain complexes of CR1-like and C3b. Residues Leu180, Trp773, Arg1320, Tyr978, Arg1082, and Val1090 at the binding interface of the human C3b–CR1 complex are all considered hotspot residues by at least two prediction tools, and these residues are conserved in porcine C3b and two CR1-like fragments. Thus, residues Leu179, Trp771, and Arg1318 of porcine C3b and residues Tyr761, Arg865, Val873, Tyr1210, Arg1314, and Val1322 of porcine CR1-like were used as active residues in restrained docking. The screening of the docking results referred to the crystal structure of the human CR1–C3b complex (PDB ID: 5FO9). The docking results showed that residue-restrained docking had a smaller RMSD value for the crystal structure of the human CR1–C3b complex compared to free docking. The docking results with the lowest RMSD values were both produced by the residue-restrained docking of HADDOCK with RMSD values of 1.795 Å and 1.797 Å, respectively. These two complexes were subjected to further molecular dynamics simulations as docking results of C3b–CR1-like SCR 12–14 and C3b–CR1-like SCR 19–21.

**Figure 4:**
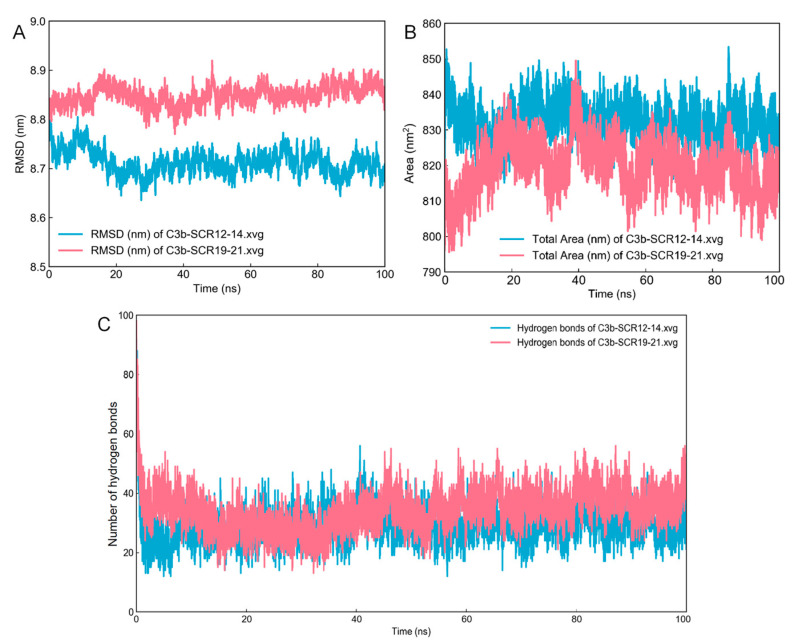
Molecular dynamics simulation of C3b–CR1-like complexes. (**A**) Backbone RMSD of C3b–CR1-like SCR 12–14 and C3b–CR1-like SCR 19–21. (**B**) Solvent accessible and surface area of C3b–CR1-like SCR 12–14 and C3b–CR1-like SCR 19–21. (**C**) Number of hydrogen bonds of C3b–CR1-like SCR 12–14 and C3b–CR1-like SCR 19–21.

Molecular dynamics simulations of 100 ns were performed for the C3b–CR1-like SCR 12–14 and C3b–CR1-like SCR 19–21 complexes, respectively, and the resulting trajectories were analyzed for stability. The RMSD of the protein backbone was plotted and analyzed to assess the extent of changes in molecular structure in the molecular dynamics’ simulation. The RMSD results showed that the C3b–CR1-like SCR 12–14 protein conformation gradually stabilized at 8.70 nm after 20 ns from the beginning of the molecular dynamics simulation, and the C3b–CR1-like SCR 19–21 protein conformation gradually stabilized at 8.85 nm after 30 ns from the beginning of the molecular dynamics simulation (Figure 4A). The solvent accessibility surface area (SASA) values of the complexes were analyzed to evaluate changes in the surface of complexes. The SASA of C3b–-CR1-like SCR 12–14 and C3b–CR1-like SCR 19–21 showed the same trend throughout the entire simulation trajectory. The SASA of both complexes showed a decreasing trend at the beginning of the simulation, then showed an increasing trend and stabilized after 50 ns at 830 nm^2^ and 820 nm^2^, respectively. This reveals that the conformational structure of the complexes reached stability during the MD simulation (Figure 4B). H-bonds have been confirmed to be an important force in maintaining the stable of complex in studies [21]. To further facilitate our understanding of the interaction between CR1-like and C3b, the gmx Hbond program was used to predict the potential number of H-bonds during the MD simulation. The number of hydrogen bonds at the binding interface of the two complexes remains essentially stable. The binding interface between C3b and CR1 SCR 19–21 contains approximately 40 hydrogen bonds, and the binding interface between C3b and CR1 SCR 12–14 contains approximately 30 hydrogen bonds (Figure 4C). 

### 2.5. Protein Interaction Analysis

**Figure 5:**
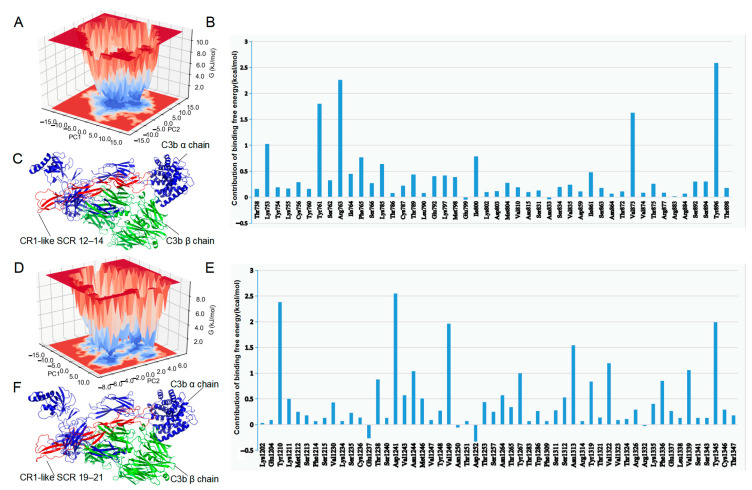
Final structure of the complex and simulated alanine mutation scan. (**A**) PCA-based free energy landscape of the C3b–CR1-like SCR 12–14 complex. (**B**) Simulated alanine mutation scan of C3b–CR1-like SCR 12–14 complex. (**C**) Final structure of the C3b–CR1-like SCR 12–14 complex. (**D**) PCA-based free energy landscape of theC3b–CR1-like SCR 19–21. (**E**) Simulated alanine mutation scan of C3b–CR1-like SCR 19–21 complex. (**F**) Final structure of the C3b–CR1-like SCR 19–21 complex.

To obtain the most stable complex conformation, a principal component analysis (PCA) was performed on the trajectory files using GROMACS, and the free energy landscape was plotted (Figure 5A,D). The final structures of C3b–CR1-like SCR 12–14 and C3b–CR1-like SCR 19–21 were the most stable protein structures extracted from the lowest energy wells of the free energy landscape map (Figure 5C,F).

The affinities and dissociation constants of C3b–CR1-like SCR 12–14 and C3b–CR1-like SCR 19–21 were predicted by PRODIGY. The affinity (ΔG) of SCR 12–14 with C3b was −12.1 kcal/mol at 25 °C and the affinity (ΔG) of SCR 19–21 for C3b was -15.3 kcal/mol at 25 °C, and the simulated alanine mutation scan of amino acid residues belonging to the CR1-like and located at the binding interface of the C3b–CR1-like complexes were performed by DrugScore^PPI^ to identify hotspot residues in the CR1-like protein. The results showed that the binding free energy of amino acids Lys753, Tyr761, Arg763, Val873, and Tyr896 of CR1-like SCR 12–14 and Tyr1210, Asp1241, Asn1244, Val1249, Tyr1267, Asn1313, Val1322, Val1339, and Tyr1345 changed by more than 1 kcal/mol after the mutation of the target amino acid to Ala (Figure 5B,E). The interaction of C3b–CR1-like was further investigated by Ligplot. In addition to the above amino acid residues, more residues were also recognized as potential binding sites based on the complementarity of the hydrophobic/hydrophilic properties of the molecular surface of the complex (Figure S5).

**Figure 6:**
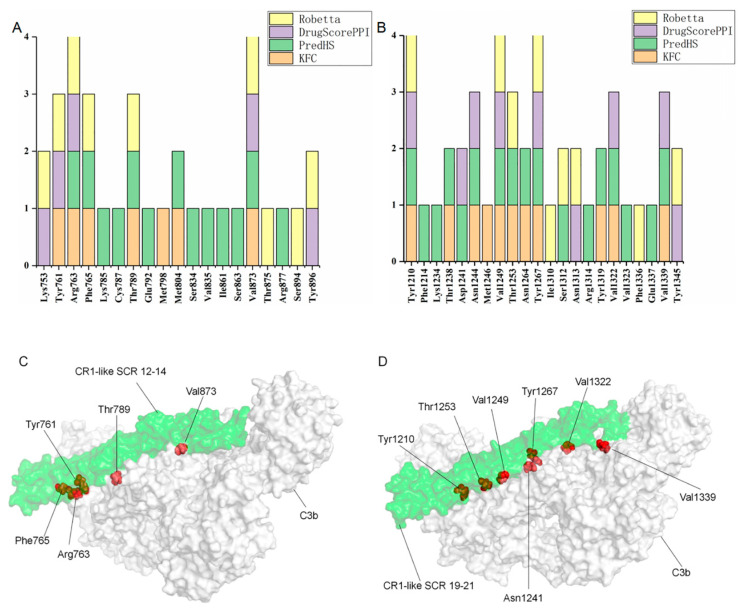
Prediction and demonstration of CR1-like hotspot residues. (**A**) Prediction of hotspot residues at the C3b–CR1-like SCR 12–14 binding interface by four prediction tools. (**B**) Prediction of hotspot residues at the C3b–CR1-like SCR 19–21 binding interface by four prediction tools. (**C**) Demonstration of C3b–CR1-like SCR 12–14 hotspot residues. (**D**) Demonstration of C3b–CR1-like SCR 19–21 hotspot residues.

To further improve the accuracy of the prediction, in addition to the DrugScore^PPI^ server, the KFC, PredHS, and Robetta servers were used for the prediction of CR1-like hotspot residues. The results showed that amino acid residues Tyr761, Arg763, Phe765, Thr789, and Val873 of SCR 12–14 and amino acid residues Tyr1210, Asn1244, Val1249, Thr1253, Tyr1267, Val1322, and Val1339 of SCR 19–21 were all considered by at least three prediction tools to be key residues involved in complex binding (Figure 6A,B). The position of the hotspot residues in the complexes are shown in Figure 6C,D.

## 3. Discussion

The immune adhesion function of erythrocytes is important for innate immunity, and porcine erythrocytes possess similar immune adhesion functions to human erythrocytes. CR1-like on the membrane surface of porcine erythrocytes is the molecular basis for their immune adhesion function. The molecular mechanisms underlying the immune adhesion function of porcine erythrocytes are not fully understood, and molecular modelling studies on porcine CR1-like binding to C3b support the clarification of the molecular mechanisms underlying the immune adhesion function of porcine erythrocytes.

Previous studies have shown that the protein sequences of SCR 8–10 and SCR 15–17 of human CR1 are identical and bind to C3b. On the other hand, the interaction of porcine CR1-like SCR 19–21 with C3b has been demonstrated by yeast two-hybrid crosses. A crystal structure analysis of the human SCR 15–17–C3b complex showed that a total of 61 amino acids of human C3b are located at the binding interface of the complex, of which 42 residues are conserved in porcine C3b. Of the predicted 12 hotspot residues in human C3b, 11 of them are conserved in porcine C3b. A total of 47 amino acids of CR1 SCR 15–17 is located at the binding interface of the complex, of which 35 residues are conserved in porcine CR1-like SCR 19–21. Of the hotspot residues among them, more than 80% are conserved on CR1-like SCR 19–21. These results indicate a high conservation of amino acids at the interface between human and porcine target proteins. Unlike human CR1, no sequences were found in the porcine CR1-like sequence that were identical to CR1-like SCR 19–21, but we found CR1-like SCR 12–14. This fragment is the most conserved fragment with CR1 SCR 15–17 except for CR1-like SCR 19–21. A total of 25 residues and over 60% of the hotspot residues at the binding interface of CR1 SCR 15–17 were conserved on porcine CR1-like SCR 12–14. This suggests that the binding interface of human CR1–C3b and porcine CR1-like–C3b is highly conserved and may have similar binding modes. We therefore selected the above two porcine CR1-like fragments for modelling to further investigate the interaction of porcine CR1-like with C3b.

AlphaFold-Multimer has good accuracy for modelling heterologous polymorphs. C3b is a protein composed of two different chains (α and β); AlphaFold-Multimer is appropriate for the homology modelling of C3b. For the modelling of the CR1-like fragment, a multiple threading approach based on I-TASSER was used as no high-quality template was available. These models were subjected to molecular dynamics simulations to optimize their structure and were evaluated for structural soundness and energy and rationality to ensure the reliability of the model structure. The optimized I-TASSER structures were compared with trRosetta, RoseTTAFold, Alphafold and human CR1 SCR 15–17 crystal structures, and the results showed that the individual structures did not differ significantly, so the model with the best structure-sequence compatibility was selected. The molecular docking of the protein was performed using blind docking and position-restricted docking, and the hotspot residues of the human CR1–C3b complex were used for the restriction amino acids. Template-based docking often generates more accurate predictions [22,23]. In molecular docking, reference is made to the human CR1-C3b co-crystal structure (5FO9), which enables more accurate docking results than blind docking. The number of RMSD, SASA, and H-bonds remained stable over a long period of time, indicating that the system had reached equilibrium in the molecular dynamics simulation and 100 ns was sufficient for the system.

The affinity of porcine C3b to CR1-like SCR12–14 and CR1-like SCR 19–21 was predicted to be -12.1 kcal/mol and -15.3 kcal/mol, respectively, after obtaining the final conformation, which is close to the affinity of human CR1 to C3b of -12 kcal/mol. Four different prediction tools were used for the prediction of hotspot residues for the two complexes. Five and seven amino acids were identified as hot residues in the porcine CR1-like SCR 12–14 and CR1-like SCR 19–21, respectively. All of these residues, excluding Thr1253 and Tyr1267, are conserved at the binding interface of human CR1 SCR 15–17-C3b. Previous mutational analysis of human CR1 showed that the simultaneous mutation of residues Lys953, Lys955, and Arg974 to Glu in SCR 15 significantly reduced the binding of CR1 to C3b [24]. However, in the eutectic structure of CR1–C3b we found that although the three residues shared a plane, they were clearly not positioned at the CR1–C3b binding interface. This suggests that the residues affecting the binding of CR1 to C3b are not only present at the binding interface but that they affect the binding of CR1 to C3b in other ways. In the corresponding region of the porcine CR1-like sequence, these residues are not fully conserved and are not at the binding interface. An analysis of this region may not be resolved via an analysis of the reciprocal model alone, and subsequent mutagenesis assays will be required to further identify key residues. Tyr978 of human CR1 is another important residue for the binding of the C3b–CR1 complex [25]. The two residues corresponding to human Tyr978 in this study were porcine Tyr761 (SCR 12–14) and Tyr1210 (SCR 19–21), both of which were identified as hotspot residues, in agreement with related human studies. The mutation of Lys1005 to Glu in human CR1 reduced C3b binding. The porcine CR1-like allelic residues were Gly788 (SCR 12–14) and Glu1237 (SCR 19–21), respectively, and the change in residues at this position was detrimental to CR1-like binding to C3b. An analysis of the CR1-like–C3b model showed that Gly788 did not contribute to CR1-like binding to C3b and that Glu1237 hindered CR1-like binding to C3b, which is consistent with mutational assays in human CR1. The Thr1080–Arg1094 region of human CR1 was shown to be associated with C3b binding [26], and the hotspot residues Val873 (SCR 12–14) and Val1322 (SCR 19–21) of porcine CR1-like in this study were both located in this region, and they were allelic residues. This study is the first to use molecular simulations to study the interaction of porcine CR1-like with C3b, and most of the experimental results are consistent with the mutational data on human CR1. The aim of this study is not to replace these experiments but to pave the way for further studies on the molecular mechanism of porcine C3b binding to porcine CR1-like, and the predicted potential hotspot residues obtained will be validated in future experiments.

## 4. Materials and Methods

### 4.1. Protein Binding Interface Analyses

The amino acid sequence of porcine CR1-like was retrieved from GenBank (GenBank Accession no. NC_010451.4). The amino acid sequence of human CR1 was retrieved from UniProtKB (Accession ID: P17927). The amino acid sequences of porcine C3b and human C3b were retrieved from UniProtKB (Accession IDs: P01025, P01024, respectively). Protein sequence alignment was performed using BLAST and Clustal Omega (https://www.ebi.ac.uk/services (accessed on 16 February 2023)) [27]. The protein domain was identified using the web server SMART (http://smart.embl.de/ (accessed on 12 October 2022)) [28,29]. Protein transmembrane regions were predicted using TMHMM 2.0 (https://services.healthtech.dtu.dk/ (accessed on 17 February 2023 )) [30]. The SignalP 6.0 server was used to detect whether any signal peptide was present in the protein [31]. We conducted protein binding interface analysis using a Python script (https://pymolwiki.org/index.php/InterfaceResidues (accessed on 12 November 2022)). We conducted hotspot residue prediction using the PredHS (http://predhs.denglab.org/ (accessed on 12 December 2022)) [32], KFC2 (https://mitchell-web.ornl.gov/KFC_Server/upload.php (accessed on 12 December 2022)) [33], Robetta (http://old.robetta.org/alascansubmit.jsp (accessed on 12 December 2022)), and DrugScore^PPI^ (https://cpclab.uni-duesseldorf.de/dsppi/main.php (accessed on 12 December 2022)) online servers [34,35].

### 4.2. Homology Modeling and Validation

All protein crystal structure templates used for homology modeling were obtained from the PDB database (https://www.rcsb.org/ (accessed on 15 October 2022)). AlphaFold-Multimer was used to predict the three-dimensional structure of C3b [36]. The trRosetta [37], RoseTTAFold [38], Alphafold [39], and I-TASSER [40] webservers (https://zhanggroup.org/I-TASSER/ (accessed on 15 October 2022)) were used to predict the three-dimensional structure of the 12–14 and 19–21 structural domains of porcine CR1-like. Five models were generated for each protein, the C3b models were scored using AlphaFold-Multimer’s weighted scoring system, and the five generated models were ranked according to their scores. The five CR1-like models correspond to the five largest structural clusters generated by I-TASSER, respectively. The confidence of each model was quantitatively measured via a C-score, which is typically in the range of (−5, 2), where a C-score of higher value signifies a model with a high confidence and vice versa. Typically, the top-ranked models have the highest C-score. The highest-scoring conformation was selected from all generated models, and the model quality was evaluated using the PROCHECK and Verify3D modules of the SAVES v6.0 webserver (https://saves.mbi.ucla.edu/ (accessed on 17 December 2022)) and the ProSA online inspection tool (https://prosa.services.came.sbg.ac.at/prosa.php (accessed on 17 December 2022)) [41,42,43]. PROCHECK analyzes the overall and residue-by-residue geometry of a protein structure, producing a Ramachandran plot that displays the φ and ψ bond angles. The plot separates the amino acids into four categories: favored, allowed, generously allowed, and disallowed. By defining a structural class depending on its position and surroundings (alpha, beta, loop, polar, and nonpolar), Verify 3D determines the compatibility of a 3D atomic model with its corresponding amino acid sequence. The tool compares its results to established structures to choose the best model. ProSA utilizes the benefits of interactive web-based applications to show scores and energy graphs, identifying possible issues in the protein structure. The software calculates a z-score, which evaluates the deviation of the total energy from an energy distribution obtained from random conformations and represents the quality of the model.

### 4.3. Molecular Dynamics Simulation of Proteins

The protein structure processing and data visualization were performed using PyMOL 4.6 and QtGrace software. Molecular dynamics (MD) simulations of the porcine CR1 and C3b models obtained from homology modeling were performed to obtain optimized models. The simulations were performed using the GROMACS version 2020.3 software package. Topologies were defined using the CHARMM36 force field [44]. The protein was accommodated in a dodecahedral box with an extension distance of 10 Å and solvated with water using the SPC 216 model. Charge neutralization of the system was achieved by adding an appropriate amount of sodium or chloride ions. The number of solvents added to the CR1-like SCR 12–14 system was 63,189, and the number of counter-ions was 3 Cl^−^. The number of solvents added to the CR1-like SCR 19–21 system was 60,721, and the number of counter-ions was 1 Cl^−^. The number of solvents added to the C3b–CR1-like SCR 12–14 system was 135,536, and the number of counter-ions was 28 Na^+^. The number of solvents added to the C3b–CR1-like SCR 19–21 system is 135,515, and the number of counter-ions was 30 Na^+^. The system was minimized by the steepest descent minimization algorithm until the maximum force <1000 kJ/mol·nm was reached. The system was equilibrated in the NVT ensemble at 300 K for 100 ps, and the system density was equilibrated in the NPT ensemble for 100 ps. After the system was equilibrated, a molecular dynamics simulation of 100 ns was performed with the step size set to 2 fs. The long-range electrostatic interactions were calculated by the particle mesh Ewald (PME) method, and the nonbonding interaction intercept was set to 1.2 nm. After the molecular dynamics simulation, root mean square deviation (RMSD) analyses were performed on the trajectory file. We extracted the stabilized trajectory file, eliminated the translation and rotation of the protein, and a perform principal component analysis on the processed trajectory. PCA was carried out in two steps; constructing a covariance matrix using Cα atoms and diagonalizing the covariance matrix. The covariance matrix was constructed from the atomic fluctuations after the removal of the translational and rotational movement. The diagonalization of this matrix yields a set of eigenvectors and eigenvalues that describe the collective modes of fluctuation of the protein. The eigenvectors corresponding to the largest eigenvalues are called “principal components”, as they represent the largest amplitude collective motions. The covariance matrix was represented in a simple linear transformation in Cartesian coordinate space [45,46]. The PCA was performed using the protocol within the GROMACS software package. First, the covariance matrix and the principal vector were calculated using the gmx covar command, after which the principal component data were generated using the gmx anaeig command. The motion of the protein was identified by projecting the first two eigenvectors (ev1 vs. ev2) containing the maximum motions. We combined the principal component 1 and principal component 2 files using the editor and used the sham command of gmx to generate the free energy landscape map. Data visualization was achieved using the XPM module of the DIT toolkit (https://github.com/CharlesHahn/DuIvyTools (accessed on 25 November 2022)). We retrieved the lowest relative energy point from the generated log file and combined it with the corresponding index file (ndx file) to obtain the corresponding frame number and output it as the final conformation. The model quality was checked again with PROCHECK and the Verify3D online tool to evaluate the model optimization effect.

### 4.4. Molecular Docking and Affinity Calculations of CR1-like and C3b

The optimized CR1-like and C3b were docked using the ClusPro, ZDOCK, FRODOCK, GRAMM-X, and HADDOCK online servers [47,48,49,50,51]. Free docking and residue-restrained docking were performed using the above five tools. The larger C3b was selected as the receptor and the smaller CR1 fragment was selected as the ligand during the docking process. Residues Leu179, Trp771, and Arg1318 of porcine C3b and residues Tyr761, Arg865, Val873, Tyr1210, Arg1314, and Val1322 of porcine CR1-like were used as active residues in residue-restrained docking. The generated docking results were imported into Pymol and compared with the crystal structure of the human CR1–C3b complex (PDB ID: 5FO9) by the align command, respectively, and the docking result with the lowest RMSD was selected. Molecular dynamics simulations of C3b–CR1-like docking complexes were carried out 100 ns to optimize the structure of the complexes. The energy minimization, temperature and pressure equilibrium, and model evaluation of the complexes were performed as described in Section 4.3. The affinity of the interacting proteins was calculated using PRODIGY [52]. The PDB file of the complex optimized by molecular dynamics simulation was imported into PRODIGY, the mode was selected as protein–protein, interactor 1 was set to the chain of C3b, interactor 2 was set to the chain of the CR1-like fragment, and the temperature was set to 25 °C.

### 4.5. Prediction of Key Amino Acids of the CR1-like-C3b Interaction

LIGPLOT was used to analyze the interaction interface between CR1-like and C3b to identify the residues involved in the interaction and the type of interaction. We selected DIMPLOT as the analysis mode, which is used to analyze protein–protein interactions. Further simulated alanine mutation scanning of complexes was performed using PredHS, KFC2, Robetta, and DrugScore^PPI^. PredHs and KFC2 are mainly used to identify hotspot residues by analyzing the physicochemical properties of the binding interface. For PredHs, the PDB file of the complex was first imported into the server, and the Query protein was set to CR1-like and Partners to C3b after the server confirmed the validity of the file. For KFC2, after importing the PDB file of the complex, Protein 1 was set to CR1-like and Protein 2 to C3b. Robetta and DrugScore^PPI^ determine key residues through the simulation of alanine scans. The mutation of CR1-like was carried out to identify key residues on CR1-like. The amino acid residues with a change of >1 kJ/mol in binding free energy after mutation were considered candidate key amino acid residue binding sites.

## 5. Conclusions

The immune adhesion function of erythrocytes is important for innate immunity, and CR1-like on the surface of porcine erythrocytes exerts its immune adhesion function by binding to C3b, but the molecular mechanism is not clear, particularly because we lack crystal structures for porcine CR1-like–C3b complexes. In this study, we constructed three-dimensional structural models of porcine C3b, CR1-like SCR 12–14, and CR1-like SCR 19–21 via homology modeling. With reference to the crystal structure of the human CR1-C3b complex, the interaction models of C3b–CR1-like SCR 12–14 and C3b–CR1-like SCR 19–21 were constructed and optimized via molecular docking and molecular dynamics simulations. The prediction of key residues for CR1-like and C3b interactions was carried out via an analysis of the binding interface. A total of 12 residues of CR1-like were considered to be potential key residues by the prediction tool, among which amino acid residues Tyr761, Arg763, Phe765, Thr789, and Val873 of SCR12–14 and amino acid residues Tyr1210, Asn1244, Val1249, Val1322, and Val1339 of SCR19–21 are conserved at the binding interface of the human C3b–CR1 SCR 15–17 complex. Among the abovementioned residues, Tyr761–Tyr1210 and Val873–Val1322 are conserved allelic residues, and their equivalents on human CR1 (Tyr978, Val1090) are hotspot residues as validated by mutagenesis assays. As a computational study, these predictions provide the basis for subsequent mutation studies, and the predicted residues will be validated preferentially.

## Figures and Tables

**Table 1:** Model optimization evaluation.

Model	Ramachandran Plot (%)				Verify 3D (%)
	Favoured	Allowed	General	Disallowed	Residues Score > 0.2%
C3b	92.2	7.7	0.1	0.1	87.2
CR1-like_(12–14)_	50.6	40.9	7.1	1.3	93.7
CR1-like_(19–21)_	72.2	23.4	3.2	1.3	95.2
CR1-like_(12–14)_^*^	85.7	12.3	1.9	0.0	81.5
CR1-like_(19–21)_^*^	88.0	10.8	1.3	0.0	89.4

* indicate the optimized protein structure.

## Data Availability

The data that support the findings of this study are available from the corresponding author upon reasonable request.

## Supplementary Materials

The following supporting information can be downloaded at: https://www.mdpi.com/article/10.3390/molecules28052183/s1, Figure S1: Sequence alignment of human CR1 SCR 15–17 fragment with the porcine CR1-like protein sequence; Figure S2: Analysis of the signal peptides and transmembrane regions of porcine CR1-like and C3b; Figure S3: Sequence alignment of human CR1-C3b binding interface with porcine CR1-like-C3b binding interface; Figure S4: Structural alignment of CR1-like models; Figure S5: Analysis of hydrogen bonding and hydrophobicity at the interaction interface. Table S1: Amino acids at the complex C3b–CR1 SCR 15–17 binding interface; Table S2: Hotspot residues at the complex C3b–CR1 SCR 15–17 binding interface; Table S3: Confidence assessment of CR1-like models generated by I-TASSER; Table S4: CR1-like model quality assessment.
