# Supplementary material for: Molecular Simulation Study on the Interaction between Porcine CR1-like and C3b
Source: Molecules. 2023 Feb 26;28(5):2183. doi: 10.3390/molecules28052183 (PMC10005376; doi:10.3390/molecules28052183)
Supplement: Supplementary file 1 [file molecules-28-02183-s001.zip › molecules-2197366-Supplementary Material.pdf]

Supplementary Material

Molecular simulation study on the interaction between porcine CR1-like and C3b

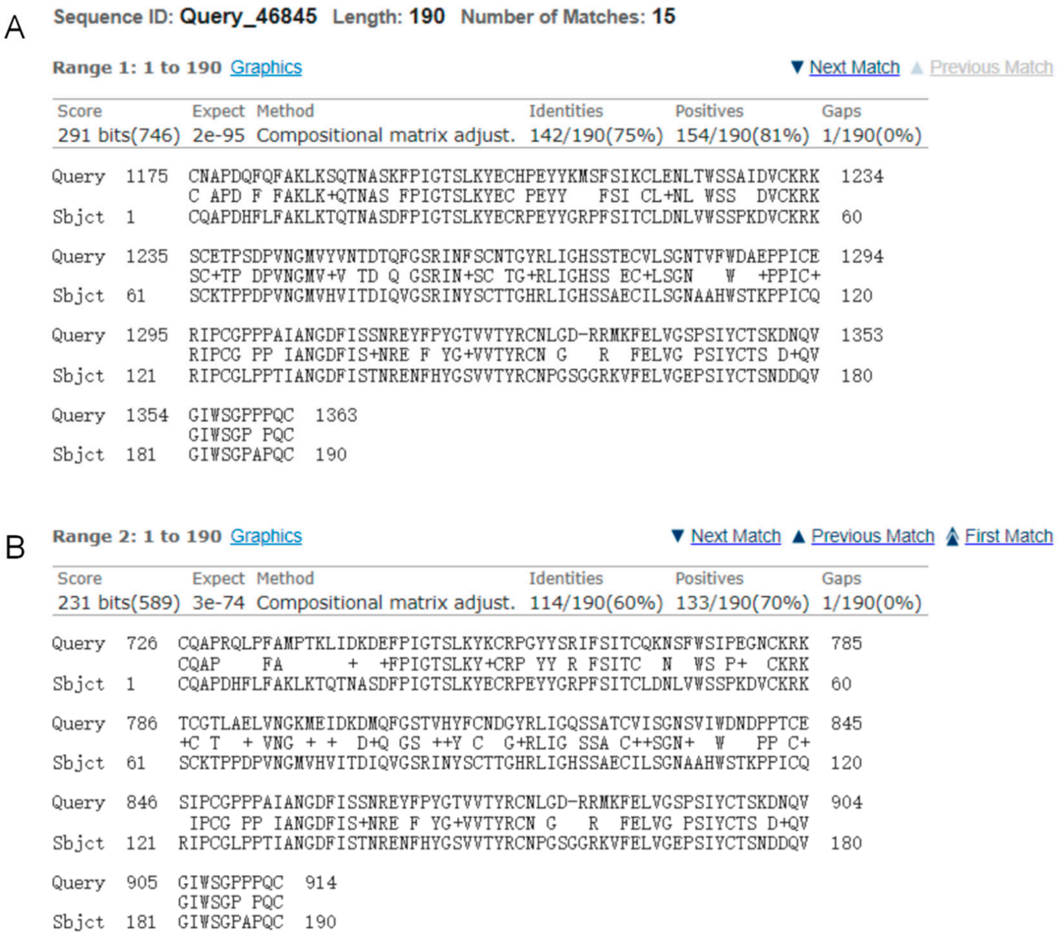

Figure S1. Sequence alignment of human CR1 SCR 15-17 fragment with the porcine CR1-like protein sequence. “Query” is porcine CR1-like protein sequence and “Sbjct” is porcine CR1-like protein sequence. (A) The fragment with the highest similarity between porcine CR1-like protein sequence and human CR1 SCR 15-17. (B) The fragment with the second highest similarity between porcine CR1-like protein sequence and human CR1 SCR 15-17.

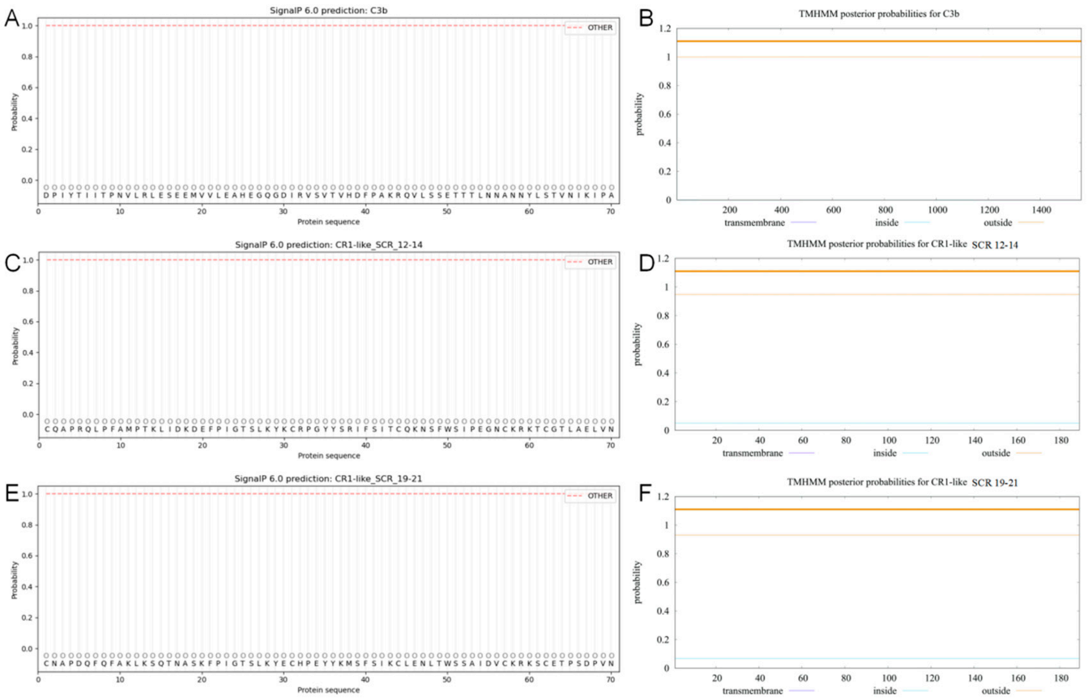

Figure S2. Analysis of the signal peptides and transmembrane regions of porcine CR1-like and C3b. (A) Signal peptide analysis of C3b. (B) Analysis of the transmembrane region of C3b. (C) Signal peptide analysis of CR1-like SCR 12-14. (D) Analysis of the transmembrane region of CR1-like SCR 12-14. (E) Signal peptide analysis of CR1-like SCR 19-21. (F) Analysis of the transmembrane region of CR1-like SCR 19-21.

A

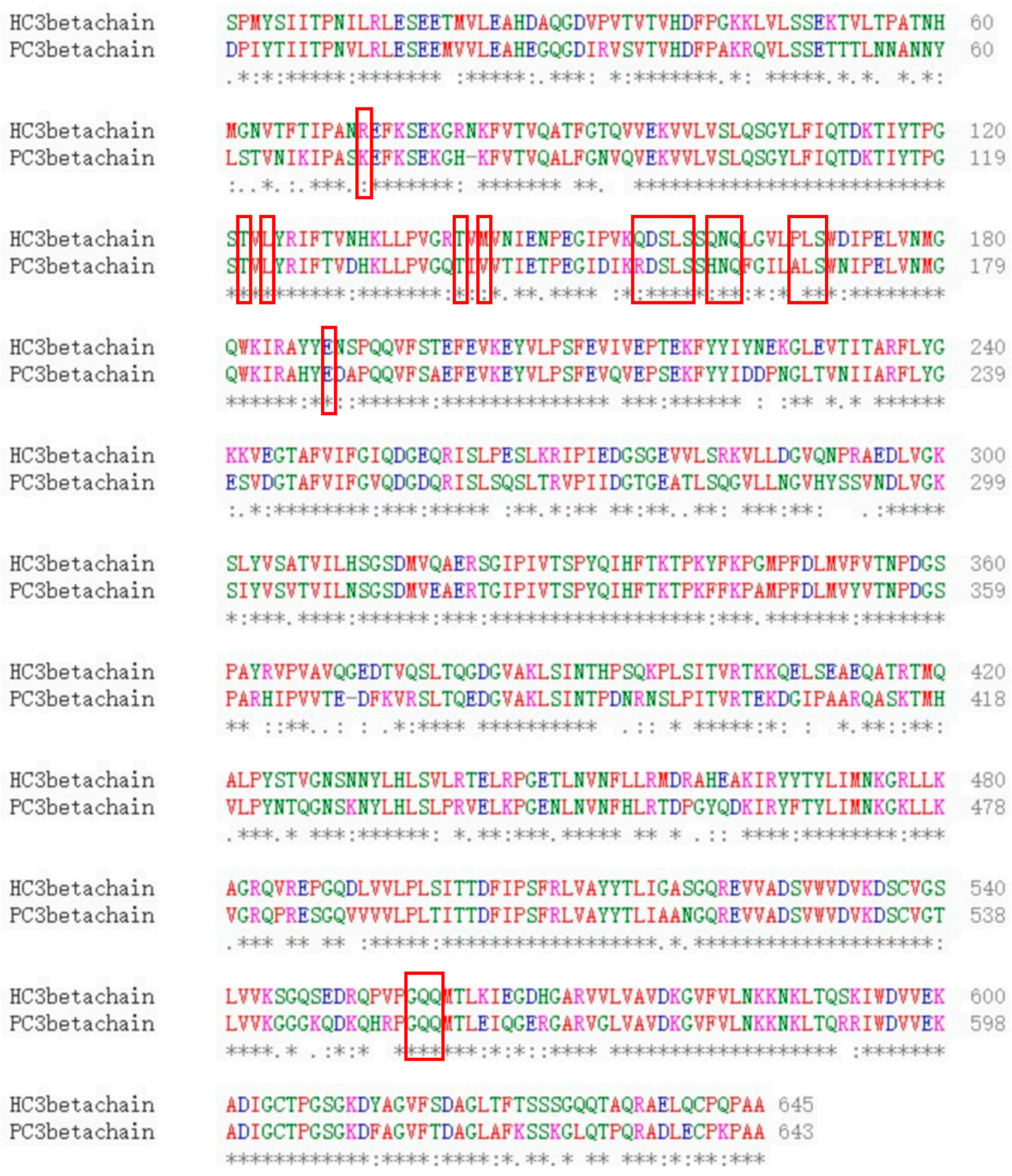
HC3betachain SPMYSIITPNILRLESEETMVLEAHDAQGDVPVTVTVHDFPGKKLVLSSEKTVLTPATNH 60
PC3betachain DPIYTIITPNVLRLESEEMVVLEAHEGQGDIRVSVTVHDFPAKRQVLSSETTTLNNANNY 60
HC3betachain MGNVTFTIPANREFKSEKGRNKFVTVQATFGTQVVEKVVLVSLQSGYLFIQTDKTIYTPG 120
PC3betachain LSTVNIKIPASKEFKSEKGH-KFVTVQALFGNVQVEKVVLVSLQSGYLFIQTDKTIYTPG 119
HC3betachain STVLYRIFTVNHKLLPVGRTVMVNIENPEGIPVKQDSLSSQNQLGVLPLSWDIPELVNMG 180
PC3betachain STVLYRIFTVDHKLLPVGQTIVVTIETPEGIDIKRDSLSSHNQFGILALSWNIPELVNMG 179
HC3betachain QWKIRAYYENSPQQVFSTEFEVKEYVLPSFEVIVEPTEKFYYIYNEKGLEVTITARFLYG 240
PC3betachain QWKIRAHYEDAPQQVFSAEFEVKEYVLPSFEVQVEPSEKFYYIDDPNGLTVNIIARFLYG 239
HC3betachain KKVEGTAFVIFGIQDGEQRISLPESLKRIPIEDGSGEVVLSRKVLLDGVQNPRAEDLVGK 300
PC3betachain ESVDGTAFVIFGVQDGDQRISLSQSLTRVPIIDGTGEATLSQGVLLNGVHYSSVNDLVGK 299
HC3betachain SLYVSATVILHSGSDMVQAERSGIPIVTSPYQIHFTKTPKYFKPGMPFDLMVFVTNPDGS 360
PC3betachain SIYVSVTVILNSGSDMVEAERTGIPIVTSPYQIHFTKTPKFFKPAMPFDLMVYVTNPDGS 359
HC3betachain PAYRVPVAVQGEDTVQSLTQGDGVAKLSINTHPSQKPLSITVRTKKQELSEAEQATRTMQ 420
PC3betachain PARHIPVVTE-DFKVRSLTQEDGVAKLSINTPDNRNSLPITVRTEKDGIPAARQASKTMH 418
HC3betachain ALPYSTVGNSNNYLHLSVLRTELRPGETLNVNFLLRMDRAHEAKIRYYTYLIMNKGRLLK 480
PC3betachain VLPYNTQGNSKNYLHLSLPRVELKPGENLNVNFHLRTDPGYQDKIRYFTYLIMNKGKLLK 478
HC3betachain AGRQVREPGQDLVVLPLSITTDFIPSFRLVAYYTLIGASGQREVVADSVWVDVKDSCVGS 540
PC3betachain VGRQPRESGQVVVVLPLTITTDFIPSFRLVAYYTLIAANGQREVVADSVWVDVKDSCVGT 538
HC3betachain LVVKSGQSEDRQPVPGQQMTLKIEGDHGARVVLVAVDKGVFVLNKKNKLTQSKIWDVVEK 600
PC3betachain LVVKGGGKQDKQHRPGQQMTLEIQGERGARVGLVAVDKGVFVLNKKNKLTQRRIWDVVEK 598
HC3betachain ADIGCTPGSGKDYAGVFSDAGLTFTSSSGQQTAQRAELQCPQPAA 645
PC3betachain ADIGCTPGSGKDFAGVFTDAGLAFKSSKGLQTPQRADLECPKPAA 643

B

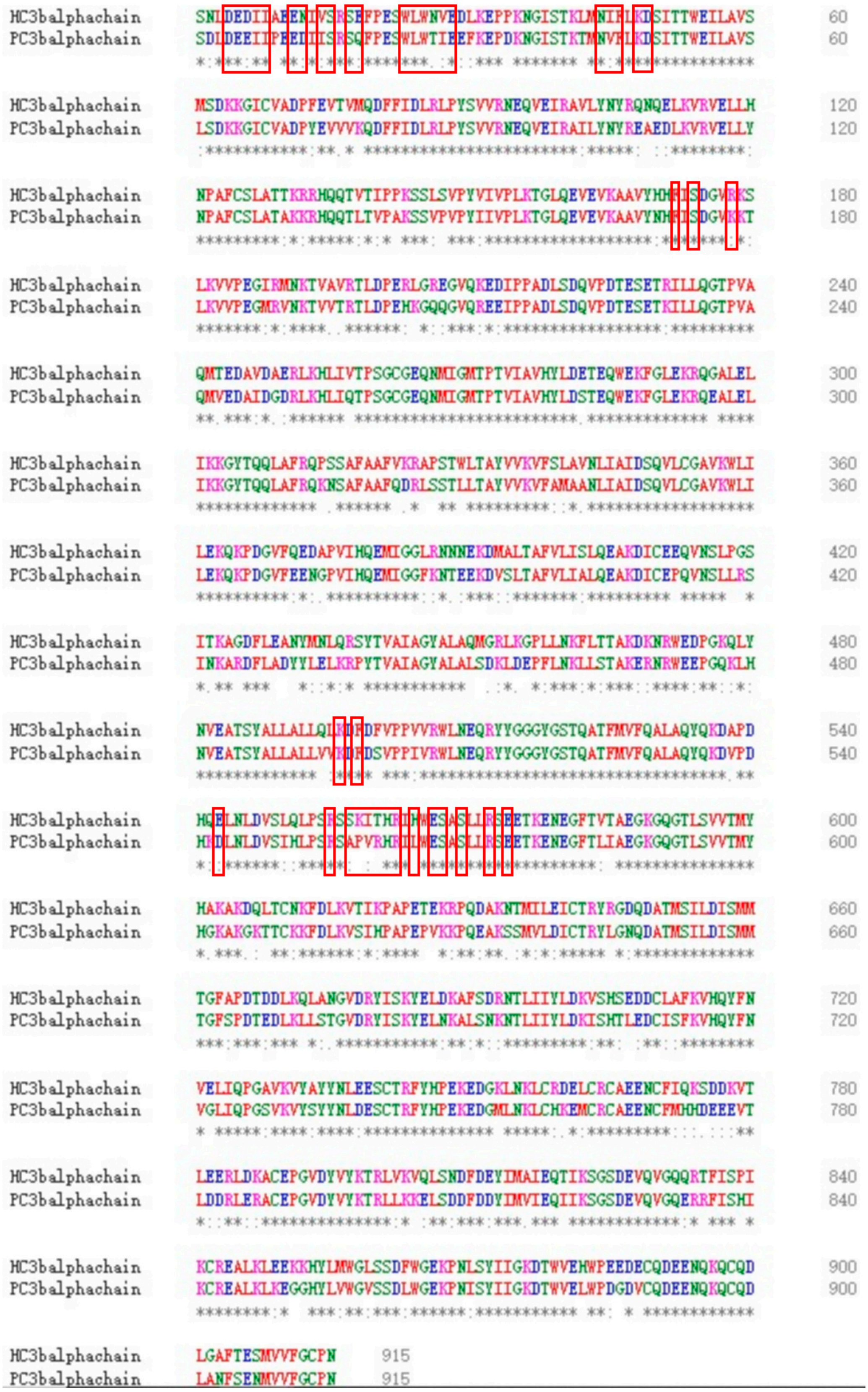
HC3balphachain SNLDEDIIAEENIVSRSEFPESWLWNVEDLKEPPKNGISTKLMNIFLKDSITTWEILAVS 60
PC3balphachain SDLDEEIIPEEDIISRSQFPESWLWTIEEFKEPDKNGISTKTMNVFLKDSITTWEILAVS 60
HC3balphachain MSDKKGICVADPFEVTVMQDFFIDLRLPYSVVRNEQVEIRAVLYNYRQNQELKVRVELLH 120
PC3balphachain LSDKKGICVADPYEVVVKQDFFIDLRLPYSVVRNEQVEIRAILYNYREAEDLKVRVELLY 120
HC3balphachain NPAFCSLATTKRRHQQTVTIPPKSSLSVPYVIVPLKTGLQEVEVKAAVYHHFISDGVRKS 180
PC3balphachain NPAFCSLATAKKRHQQTLTVPAKSSVPVPYIIVPLKTGLQEVEVKAAVYNHFISDGVKKT 180
HC3balphachain LKVVPEGIRMNKTVAVRTLDPERLGREGVQKEDIPPADLSDQVPDTESETRILLQGTPVA 240
PC3balphachain LKVVPEGMRVNKTVVTRTLDPEHKGQQGVQREEIPPADLSDQVPDTESETKILLQGTPVA 240
HC3balphachain QMTEDAVDAERLKHLIVTPSGCGEQNMIGMTPTVIAVHYLDETEQWEKFGLEKRQGALEL 300
PC3balphachain QMVEDAIDGDRLKHLIQTPSGCGEQNMIGMTPTVIAVHYLDSTEQWEKFGLEKRQEALEL 300
HC3balphachain IKKGYTQQLAFRQPSSAFAAFVKRAPSTWLTAYVVKVFSLAVNLIAIDSQVLCGAVKWLI 360
PC3balphachain IKKGYTQQLAFRQKNSAFAAFQDRLSSTLLTAYVVKVFAMAANLIAIDSQVLCGAVKWLI 360
HC3balphachain LEKQKPDGVFQEDAPVIHQEMIGGLRNNNEKDMALTAFVLISLQEAKDICEEQVNSLPGS 420
PC3balphachain LEKQKPDGVFEENGPVIHQEMIGGFKNTEEKDVSLTAFVLIALQEAKDICEPQVNSLLRS 420
HC3balphachain ITKAGDFLEANYMNLQRSYTVAIAGYALAQMGRLKGPLLNKFLTTAKDKNRWEDPGKQLY 480
PC3balphachain INKARDFLADYYLELKRPYTVAIAGYALALSDKLDEPFLNKLLSTAKERNRWEEPGQKLH 480
HC3balphachain NVEATSYALLALLQLKDFDFVPPVVRWLNEQRYYGGGYGSTQATFMVFQALAQYQKDAPD 540
PC3balphachain NVEATSYALLALLVVKDFDSVPPIVRWLNEQRYYGGGYGSTQATFMVFQALAQYQKDVPD 540
HC3balphachain HQELNLDVSLQLPSRSSKITHRIHWESASLIRSEETKENEGFTVTAEGKGQGTLSVVTMY 600
PC3balphachain HKDLNLDVSIHLPSRSAPVRHRILWESASLIRSEETKENEGFTLIAEGKGQGTLSVVTMY 600
HC3balphachain HAKAKDQLTCNKFDLKVTIKPAPETEKRPQDAKNTMILEICTRYRGDQDATMSILDISMM 660
PC3balphachain HGKAKGKTTCKKFDLKVSIHPAPEPVKKPQEAKSSMVLDICTRYLGNQDATMSILDISMM 660
HC3balphachain TGFAPDTDDLKQLANGVDRYISKYELDKAFSDRNTLIIYLDKVSHSEDDCLAFKVHQYFN 720
PC3balphachain TGFSPDTEDLKLLSTGVDRYISKYELNKALSNKNTLIIYLDKISHTLEDCISFKVHQYFN 720
HC3balphachain VELIQPGAVKVYAYYNLEESCTRFYHPEKEDGKLNKLCRDELCRCAEENCFIQKSDDKVT 780
PC3balphachain VGLIQPGSVKVYSYYNLDESCTRFYHPEKEDGMLNKLCHKEMCRCAEENCFMHHDEEEVT 780
HC3balphachain LEERLDKACEPGVDYVYKTRLVKVQLSNDFDEYIMAIEQTIKSGSDEVQVGQQRTFISPI 840
PC3balphachain LDDRLERACEPGVDYVYKTRLLKKELSDDFDDYIMVIEQIIKSGSDEVQVGQERRFISHI 840
HC3balphachain KCREALKLEEKKHYLMWGLSSDFWGEKPNLSYIIGKDTWVEHWPEEDECQDEENQKQCQD 900
PC3balphachain KCREALKLKEGGHYLVWGVSSDLWGEKPNISYIIGKDTWVELWPDGDVCQDEENQKQCQD 900
HC3balphachain LGAFTESMVVFGCPN 915
PC3balphachain LANFSENMVVFGCPN 915

C

```
PCR112-14    CQAPRQLPFAMPTKLIDKDEFPIGTSLKYKCRPGYYSRIFSITCQKNSFWSIPEGNCKRK    60
HCR115-17    CQAPDHFLFAKLKTQTNASDFPIGTSLKYECRPEYYGRPFSITCLDNLVWSSPKDVCKRK    60
PCR119-21    CNAPDQFQFAKLKSQTNASKFPIGTSLKYECHPEYYKMSFSIKCLENLTWSSAIDVCKRK    60
             *:** :: **  ..  : ..*********:*:* **   ***.* .*  **   . ****

PCR112-14    TCGTLAELVNGKMEIDKDMQFGSTVHYFCNDGYRLIGQSSATCVISGNSVIWDNDPPTCE    120
HCR115-17    SCKTPPDFVNGMVHVITDIQVGSRINYSCTTGHRLIGHSSAECILSGNTAHWSTKPPICQ    120
PCR119-21    SCETPSDFVNGMVYVNTDTQFGSRINFSCNTGYRLIGHSSTECVLSGNTVFWDAEPPICE    120
             :* *  : ***  : : .* *.** ::: *. *:****:**: *::***:. *. .** *:

PCR112-14    SIPCGPPPAIANGDFISSNREYFPYGTVMTYRCNLGDRR-MKFELVGSPSIYCTSKDNQV    179
HCR115-17    RIPCGLPPTIANGDFISTNRENFHYGSVMTYRCNLGSRGRKVFELVGEPSIYCTSNDDQV    180
PCR119-21    RIPCGPPPAIANGDFISSNREYFPYGTVMTYRCNLGDRR-MKFELVGSPSIYCTSKDNQV    179
              **** **:********:*** * **:*********.*    *****.*******:*:**

PCR112-14    GIWSGPPPQC    189
HCR115-17    GIWSGPAPQC    190
PCR119-21    GIWSGPPPQC    189
             ****** ***
```

Figure S3. Sequence alignment of human CR1-C3b binding interface with porcine CR1-like-C3b binding interface. The residues enclosed in the red box are the residues located at the interface of human CR1-C3b complex and the equivalent residues in porcine CR1-like-C3b. The order of residues corresponds to Table S1. “*” indicates positions that have a single, fully conserved residue. “:” indicates that one of the subsequent 'strong' groups is fully conserved. “.” indicates that one of the subsequent ‘weaker’ groups is fully conserved. “H” stands for human, “P” stands for porcine. (A) Sequence alignment of human C3b β chain (23-667) and porcine C3b β chain (23-665). (B) Sequence alignment of human C3b α chain (749-1663) and porcine C3b α chain (747-1661). (C) Sequence alignment of human CR1 SCR 15-17 (943-1132), porcine CR1-like SCR 12-14 (726-914) and porcine CR1-like SCR 19-21 (1175-1363).

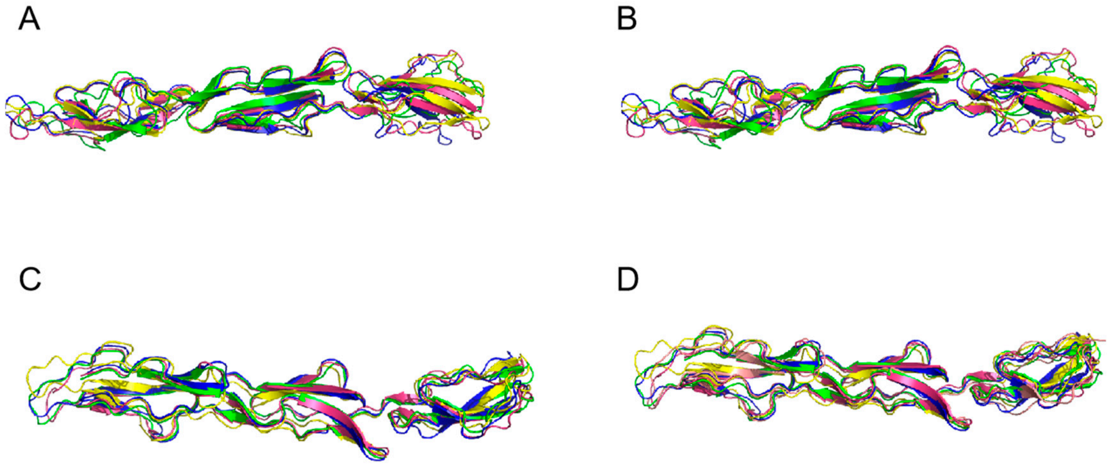

Figure S4. Structural alignment of CR1-like models. The human CR1 crystal structure is pink. ITASSER CR1-like models generated by I-TASSER, trRosetta, RoseTTAFold and Alphafold are yellow, green, warm pink and blue respectively. (A) Structural comparison of trRosetta, RoseTTAFold and Alphafold with ITASSER generated models (CR1-like SCR 12-14). (B) Structural comparison of models (CR1-like SCR 12-14) generated by trRosetta, RoseTTAFold, Alphafold and ITASSER with human CR1 SCR 15-17. (C) Structural comparison of trRosetta, RoseTTAFold and Alphafold with ITASSER generated models (CR1-like SCR 19-21). (D) Structural comparison of models (CR1-like SCR 19-21) generated by trRosetta, RoseTTAFold , Alphafold and ITASSER with human CR1 SCR 15-17.

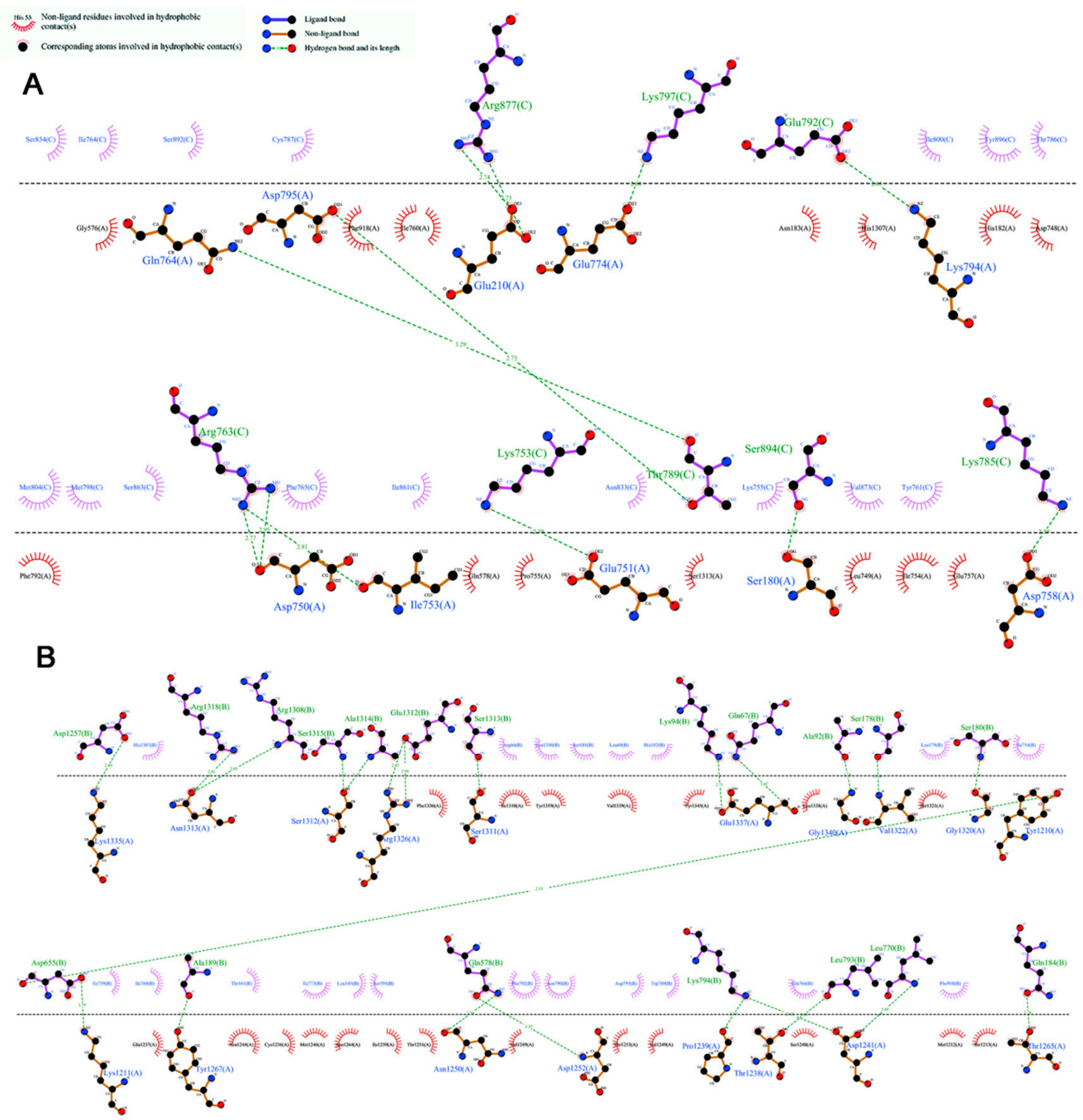

Figure S5. Analysis of hydrogen bonding and hydrophobicity at the interaction interface. (A) LIGPLOT analysis of the C3b–CR1-like SCR 12-14 complex. (B) LIGPLOT analysis of the C3b－CR1-like SCR 19-21 complex.

Table S1. Amino acids at the complex C3b – CR1 SCR 15-17 binding interface

Protein	Residue	Chain/domain
C3b	Arg94	C3b beta chain
C3b	Thr144	C3b beta chain
C3b	Leu146	C3b beta chain
C3b	Thr162	C3b beta chain
C3b	Met164	C3b beta chain
C3b	Gln177	C3b beta chain
C3b	Asp178	C3b beta chain
C3b	Ser179	C3b beta chain
C3b	Leu180	C3b beta chain
C3b	Ser181	C3b beta chain
C3b	Gln183	C3b beta chain
C3b	Asn184	C3b beta chain
C3b	Gln185	C3b beta chain
C3b	Asn190	C3b beta chain
C3b	Leu191	C3b beta chain
C3b	Ser192	C3b beta chain
C3b	Glu211	C3b beta chain
C3b	Gly578	C3b beta chain
C3b	Gln579	C3b beta chain
C3b	Gln580	C3b beta chain
C3b	Asp752	C3b alpha chain
C3b	Glu753	C3b alpha chain
C3b	Asp754	C3b alpha chain
C3b	Ile755	C3b alpha chain
C3b	Ile756	C3b alpha chain
C3b	Glu759	C3b alpha chain
C3b	Asn760	C3b alpha chain
C3b	Val762	C3b alpha chain
C3b	Ser763	C3b alpha chain
C3b	Ser765	C3b alpha chain
C3b	Glu766	C3b alpha chain
C3b	Trp771	C3b alpha chain
C3b	Leu772	C3b alpha chain
C3b	Trp773	C3b alpha chain
C3b	Asn774	C3b alpha chain
C3b	Val775	C3b alpha chain
C3b	Glu776	C3b alpha chain
C3b	Asn792	C3b alpha chain
C3b	Ile793	C3b alpha chain
C3b	Phe794	C3b alpha chain

C3b	Lys796	C3b alpha chain
C3b	Asp797	C3b alpha chain
C3b	Phe920	C3b alpha chain
C3b	Ser922	C3b alpha chain
C3b	Arg926	C3b alpha chain
C3b	Lys1244	C3b alpha chain
C3b	Phe1246	C3b alpha chain
C3b	Glu1291	C3b alpha chain
C3b	Arg1303	C3b alpha chain
C3b	Ser1305	C3b alpha chain
C3b	Lys1306	C3b alpha chain
C3b	Ile1307	C3b alpha chain
C3b	Thr1308	C3b alpha chain
C3b	His1309	C3b alpha chain
C3b	Arg1310	C3b alpha chain
C3b	His1312	C3b alpha chain
C3b	Glu1314	C3b alpha chain
C3b	Ser1315	C3b alpha chain
C3b	Ser1317	C3b alpha chain
C3b	Arg1320	C3b alpha chain
C3b	Glu1322	C3b alpha chain
CR1	Tyr978	SCR 15-17
CR1	Arg980	SCR 15-17
CR1	Asp997	SCR 15-17
CR1	Val998	SCR 15-17
CR1	Lys1000	SCR 15-17
CR1	Lys1002	SCR 15-17
CR1	Ser1003	SCR 15-17
CR1	Lys1005	SCR 15-17
CR1	Thr1006	SCR 15-17
CR1	Asn1007	SCR 15-17
CR1	Asn1008	SCR 15-17
CR1	Asp1009	SCR 15-17
CR1	Val1011	SCR 15-17
CR1	Asn1012	SCR 15-17
CR1	Gly1013	SCR 15-17
CR1	Met1014	SCR 15-17
CR1	Val1015	SCR 15-17
CR1	His1016	SCR 15-17
CR1	Val1017	SCR 15-17
CR1	Thr1019	SCR 15-17
CR1	Asp1020	SCR 15-17
CR1	Ile1021	SCR 15-17
CR1	Thr1032	SCR 15-17

CR1	Thr1033	SCR 15-17
CR1	His1035	SCR 15-17
CR1	Asn1050	SCR 15-17
CR1	Thr1051	SCR 15-17
CR1	Asp1076	SCR 15-17
CR1	Ile1078	SCR 15-17
CR1	Ser1079	SCR 15-17
CR1	Thr1080	SCR 15-17
CR1	Asn1081	SCR 15-17
CR1	Arg1082	SCR 15-17
CR1	Glu1083	SCR 15-17
CR1	Asn1084	SCR 15-17
CR1	His1086	SCR 15-17
CR1	Tyr1087	SCR 15-17
CR1	Gly1088	SCR 15-17
CR1	Ser1089	SCR 15-17
CR1	Val1090	SCR 15-17
CR1	Thr1092	SCR 15-17
CR1	Arg1094	SCR 15-17
CR1	Ser1099	SCR 15-17
CR1	Arg1100	SCR 15-17
CR1	Ser1112	SCR 15-17
CR1	Tyr1114	SCR 15-17
CR1	Thr1116	SCR 15-17

Table S2. Hotspot residues at the complex C3b－CR1 SCR 15-17 binding interface

Protein	Residue	Chain/domain	KFC	PredHS	DrugScore^{PPI}
C3b	Asp178	C3b beta chain	+	-	X
C3b	Ser179	C3b beta chain	+	-	X
C3b	Leu180	C3b beta chain	+	+	X
C3b	Ser181	C3b beta chain	-	+	X
C3b	Gln185	C3b beta chain	-	+	X
C3b	Val762	C3b alpha chain	+	-	X
C3b	Trp773	C3b alpha chain	+	+	X
C3b	Phe794	C3b alpha chain	+	-	X
C3b	Lys796	C3b alpha chain	+	-	X
C3b	Phe920	C3b alpha chain	+	-	X
C3b	His1309	C3b alpha chain	+	-	X
C3b	Arg1320	C3b alpha chain	+	+	X
CR1	Tyr978	SCR 15-17	+	-	+
CR1	Arg980	SCR 15-17	-	+	-
CR1	Thr1006	SCR 15-17	+	-	-
CR1	Asp1009	SCR 15-17	+	-	-
CR1	Asn1012	SCR 15-17	-	+	-
CR1	Met1014	SCR 15-17	-	+	-
CR1	Val1017	SCR 15-17	+	-	-
CR1	Thr1080	SCR 15-17	+	+	-
CR1	Arg1082	SCR 15-17	+	-	+
CR1	Val1090	SCR 15-17	+	+	+
CR1	Tyr1114	SCR 15-17	-	-	+

‘+’ indicates a positive result, ‘-’ indicates a negative result, ‘X’ indicates that no analysis was performed.

Table S3. Confidence assessment of CR1-like models generated by I-TASSER.

Model	C-score	TM-score	Estimated RMSD
SCR12-14	-1.13	0.57±0.14	7.7±4.3Å
SCR 19-21	-0.42	0.66±0.13	6.1±3.8Å

Table S4. CR1-like model quality assessment.

Model	Tools	Ramachandran plot (%)				Verify 3D (%)
		Favoured	Allowed	General	Disallowed	residues score>0.2%
CR1-like$_{(12-14)}$	I-TASSER	85.7	12.3	1.9	0.0	81.5
CR1-like$_{(12-14)}$	trRosetta	89.0	9.7	0.6	0.6	28.0
CR1-like$_{(12-14)}$	RoseTTAFold	88.3	10.4	0.6	0.6	32.8
CR1-like$_{(12-14)}$	Alphafold	90.3	9.7	0.0	0.0	31.2
CR1-like$_{(19-21)}$	I-TASSER	88.0	10.8	1.3	0.0	89.4
CR1-like$_{(19-21)}$	trRosetta	89.2	10.1	0.0	0.6	25.4
CR1-like$_{(19-21)}$	RoseTTAFold	88.0	10.8	0.0	1.3	35.5
CR1-like$_{(19-21)}$	Alphafold	87.3	12.7	0.0	0.0	29.6
